# A Case of Megaesophagus Secondary to a Massive Phytobezoar in a Patient With Achalasia

**DOI:** 10.7759/cureus.23061

**Published:** 2022-03-11

**Authors:** Syed Salman Hamid Hashmi, Jennifer Dimino, Ahmed Shady, Jennifer Harley, Ashley Maranino

**Affiliations:** 1 Internal Medicine, NYU (New York University) Langone/Woodhull Hospital, New York, USA; 2 Gastroenterology, New York Medical College, Metropolitan Hospital Center, New York, USA

**Keywords:** upper endoscopy, diet pepsi lavage, mega-esophagus, severe achalasia, phytobezoar

## Abstract

Bezoar is a rare entity that is composed of indigested foreign material and is most commonly seen in the stomach. Phytobezoars are the most common type of bezoars and are composed of indigestible cellulose and lignin from fruits and vegetables. We present a unique case of esophageal phytobezoar, which was seen in a patient with long-standing achalasia. The patient presented to the gastroenterology clinic complaining of decreased appetite as she had worsening dysphagia, weight loss, vomiting on eating food. An endoscopy revealed a large phytobezoar that was extending along the whole length of the esophagus. There was stenosis at the gastroesophageal (GE) junction. The phytobezoar was dissolved with carbonated soda lavage and the remainder of the phytobezoar was fragmented with water irrigation and rescue net via the endoscope and fragments were retrieved. Botulinum was injected at the GE junction in all four quadrants which resulted in a relaxation of the stenosis. Untreated long-standing esophageal phytobezoars can lead to life-threatening complications like perforation. Endoscopic modalities with carbonated soda lavage is an efficacious mode of treatment. Surgical interventions are recommended in case of endoscopic modality failure.

## Introduction

Bezoars are a rare clinical entity seen in 0.4% of the general population and are indigested foreign material that can be located anywhere in the gastrointestinal (GI) tract. They are most commonly found in the stomach but esophageal bezoars are very rare. Impaired gastric dysmotility, low gastric acidity, connective tissue disorders, prior gastrointestinal surgeries are the predisposing factor for bezoar formation [1,2]. Phytobezoars are the most common type of bezoars, which are composed of indigestible cellulose and lignin from fruits and vegetables. Patients with phytobezoars may present with epigastric discomfort, nausea, vomiting, early satiety, weight loss, dysphagia, or upper gastrointestinal ulceration and hemorrhage. Phytobezoars were typically treated with surgery before 1970 and since then a wide variety of therapeutic options have been reported which include endoscopic removal using papain, cellulase, or N-acetylcysteine [1,2,3]. One such interesting treatment was the successful use of carbonated soda to dissolve phytobezoars. In this case report, we present a case of a complicated phytobezoar that extended along the whole length of the esophagus, which was fragmented and dissolved using a carbonated soda lavage via endoscopic interventions. 

## Case presentation

An 82-year-old female with a past medical history of achalasia type 2 was admitted for a surveillance endoscopy for Los Angeles (LA) class D esophagitis. Six years ago, the patient had presented with weight loss, nausea, vomiting, and difficulty eating solid food and a CT scan of the thorax revealed a suspected mid-esophageal mass. Further workup with a barium swallow esophagography revealed a bird's beak appearance of the lower esophageal sphincter (LES). A manometric study revealed inadequate LES relaxation in response to wet swallows, confirming the diagnosis of achalasia. Upper endoscopy revealed a mid-esophageal phytobezoar, which was dissolved using a carbonated soda lavage, and the underlying esophagus showed esophagitis. The patient refused any surgical interventions for the treatment of achalasia. After her first endoscopy, the patient was discharged on a soft diet but due to poor compliance with recommended diet, she presented to the gastroenterology clinic a year later with complaints of anorexia, worsening dysphagia, weight loss, and emesis upon eating food.

**Figure 1:**
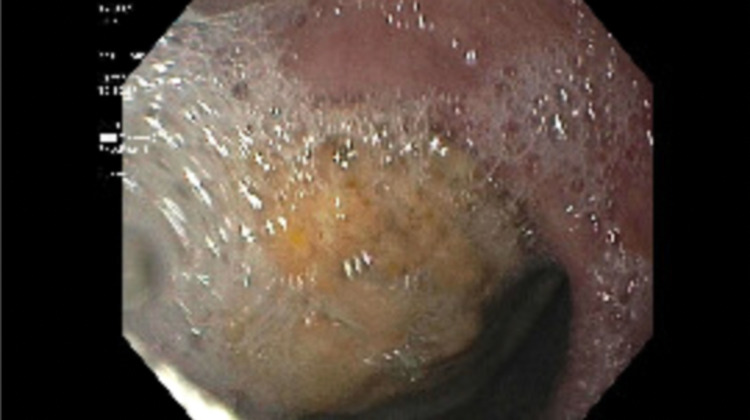
Upper endoscopy. The image shows the upper third of the esophagus with more than 90% occlusion with the phytobezoar.

**Figure 2:**
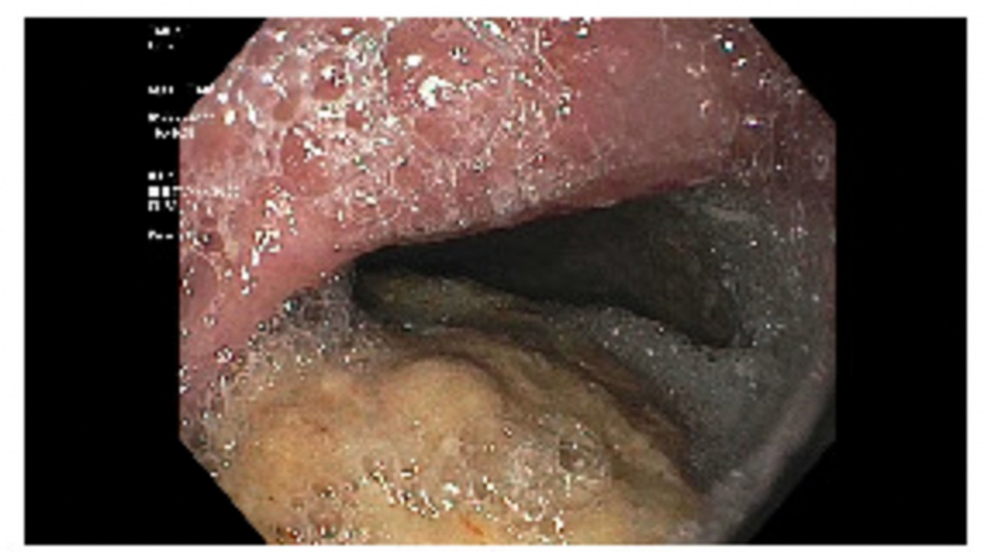
Upper endoscopy. The image shows the middle third of the esophagus occluded with the phytobezoar.

**Figure 3:**
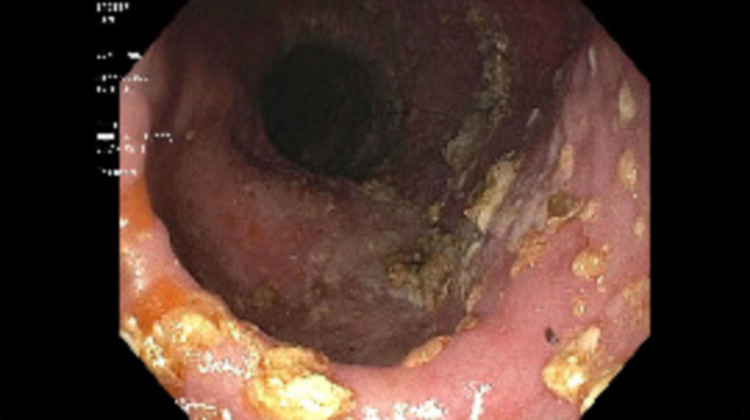
Upper endoscopy. The image shows the dissolution of the phytobezoar after carbonated soda lavage revealing Los Angeles grade D esophagitis.

**Figure 4:**
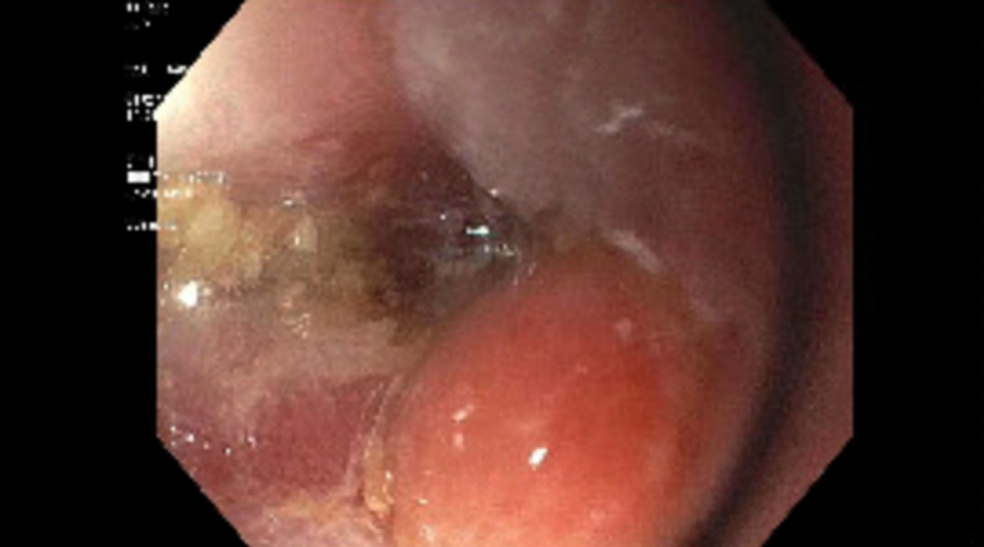
Upper endoscopy. The image shows stenosis at the gastroesophageal junction.

**Figure 5:**
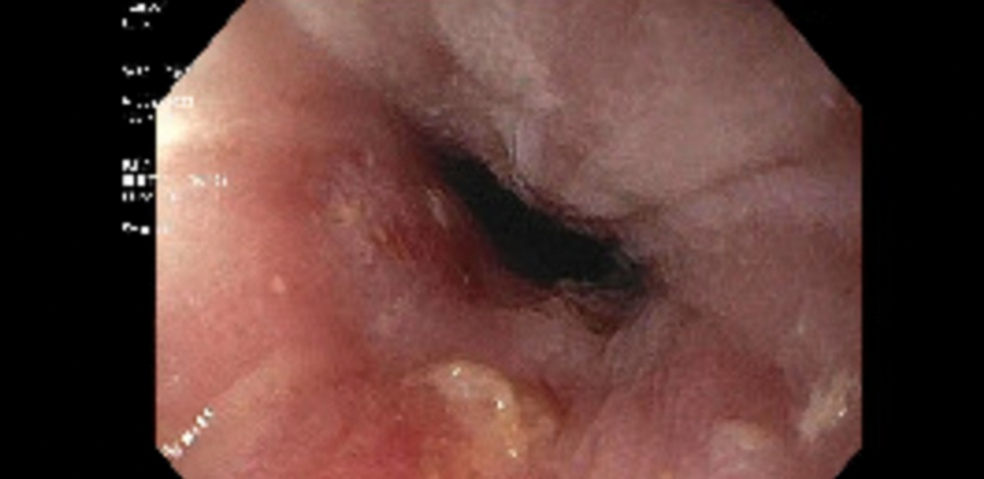
Upper endoscopy. The image shows relaxation of stenosis after botulinum injection in all four quadrants of the gastroesophageal junction.

During the surveillance endoscopy, a large phytobezoar was encountered immediately upon passing the upper esophageal sphincter (UES). The phytobezoar occluded more than 90% of the lumen (Figure 1) and the endoscope could not be advanced past the upper esophageal sphincter. The patient was intubated for airway protection from food impaction. After intubation, the endoscope was passed and at the level of the upper esophageal sphincter, a lavage using carbonated soda was performed resulting in the partial dissolution of the phytobezoar at the UES. The endoscope was advanced and it revealed a large phytobezoar that was blocking the esophagus causing increased esophageal diameter. A reminder of the phytobezoar along the whole length of the esophagus (Figure. 2) was dissolved using the carbonated soda lavage and fragmented with water irrigation resulting in the dissolution of the phytobezoar and the fragments were retrieved using a rescue net (Figure. 3). Close to 300 ml of carbonated soda was used in the lavage. There was LA grade D esophagitis without bleeding and stenosis was encountered at the GE junction, which was traversed with mild resistance (Figure. 4). Twenty units each of botulinum was injected in all four quadrants of the GE junction, which resulted in a relaxation of the stenosis and the scope could be re-traversed with minimal resistance (Figure. 5). The endoscopy also revealed gastritis in the gastric body and antrum with a normal duodenum. After the procedure, the patient was instructed to comply with a liquid diet to prevent further impaction. An extensive discussion was done with the patient and the family who agreed to consider advanced modalities like per-oral endoscopic myotomy (POEM) for the treatment of achalasia.

## Discussion

Bezoars occur in critically ill patients on mechanical ventilator therapy or in long-term bedridden patients receiving enteral feed via a nasogastric tube [2]. Phytobezoars typically develop in patients with lower esophageal sphincter dysfunction, achalasia, and progressive systemic sclerosis with a distal esophageal stricture [1,2,3]. Endoscopic interventions remain the mainstay for the diagnosis and treatment of esophageal bezoars.

Management of phytobezoars involves dissolution, fragmentation, and retrieval of the material. The risk of aspiration from fragments during the intervention makes bezoars in the esophagus demanding to treat and most of the interventions are performed using overtube-assisted endoscopy [3]. Therapeutic management can be challenging, especially for the phytobezoars extending the length of the esophagus, and treatment modalities include endoscopic interventions and lavage with a carbonated soda and pancreatic enzymes like lipase. In a study by Marcelo et al., 91.3%, of phytobezoars were successfully treated using carbonated soda and endoscopic interventions [1]. A regimen of carbonated soda and cellulase preparation has also been used successfully to dissolve a phytobezoar [3]. The mechanism of action of carbonated soda for the dissolution of phytobezoars is not certain but is hypothesized that the sodium bicarbonate acts as a mucolytic and the carbon dioxide penetrates the bezoar which is believed to digest the fibers [3]. 

Of the patients with longstanding achalasia, 10-15% have aperistalsis of the esophagus and have repeated bezoar formation. Such patients do not respond to therapeutic procedures and they benefit from POEM and close to 5% of patients will end up needing surgical intervention [1,4,5,6,7]. In our patient, we successfully dissolved a large phytobezoar that was occluding more than 90% of the lumen of the esophagus using carbonated soda lavage with endoscopic interventions and the stenosis at the GE junction also responded appropriately to the botulinum injections. 

## Conclusions

Esophageal phytobezoars are a rare entity that is most commonly seen in patients with esophageal dysmotility and can cause megaesophagus similar to the case presentation. Long-standing achalasia can cause life-threatening complications like perforation and aspiration. Endoscopic modalities and the use of carbonated soda lavage are the mainstays of treatment. Surgical interventions are recommended in case of endoscopic modality failure. In patients with achalasia, lifestyle modification like a soft diet and consumption of an adequate amount of fluid might help to prevent phytobezoar. Though carbonated soda has been used to dissolve phytobezoars, there is no research evidence showing that it prevents phytobezoar formation in patients with esophageal dysmotility. 

## References

[1] Vela MF, Richter JE, Wachsberger D, Connor J, Rice TW (2004). Complexities of managing achalasia at a tertiary referral center: use of pneumatic dilatation, Heller myotomy, and botulinum toxin injection. Am J Gastroenterol.

[2] Liang JJ, Murray JA (2016). Esophageal bezoar in the setting of achalasia. Dis Esophagus.

[3] Kim KH, Choi SC, Seo GS, Kim YS, Choi CS, Im CJ (2010). Esophageal bezoar in a patient with achalasia: case report and literature review. Gut Liver.

[4] Duranceau A, Liberman M, Martin J, Ferraro P (2012). End-stage achalasia. Dis Esophagus.

[5] Goel AK, Seenu V, Srikrishna NV, Goyal S, Thakur KK, Shukla NK (1995). Esophageal bezoar: a rare but distinct clinical entity. Trop Gastroenterol.

[6] Grosskopf I, Streifler J, Garty M, Rosenfeld JB (1986). Esophageal bezoar in progressive systemic sclerosis. J Clin Gastroenterol.

[7] Okamoto Y, Yamauchi M, Sugihara K, Kato H, Nagao M (2007). Is coca-cola effective for dissolving phytobezoars?. Eur J Gastroenterol Hepatol.

